# Effects of Parity and Somatic Cell Count Threshold on Udder Morphology, Milkability Traits, and Milk Quality in Canarian Goats

**DOI:** 10.3390/ani14091262

**Published:** 2024-04-23

**Authors:** Mario Salomone-Caballero, María Fresno, Sergio Álvarez, Alexandr Torres

**Affiliations:** Unit of Animal Production, Pasture, and Forage in Arid and Subtropical Areas, Canary Islands Institute for Agricultural Research (ICIA), 28260 Tenerife, Spain; mario.salomone@gmail.com (M.S.-C.); mfresno@icia.es (M.F.); salvarez@icia.es (S.Á.)

**Keywords:** dairy goat, milk flow, parity, somatic cell count, udder morphology

## Abstract

**Simple Summary:**

Parity can affect milk yield, milk emission kinetics, and somatic cell count in milk (SCC) in dairy animals, because the mammary gland plays an important role in milk storage capacity between primiparous and multiparous animals. On the other hand, European regulations provide specific SCC limits for cow milk due to it is a well-established indicator of udder health, but lack such criteria for goat or sheep milk. This research paper addresses the effects of parity and SCC threshold on the udder morphology, milkability traits, and milk composition during mid-lactation in Canarian goats. Results showed a positive association between SCC and total bacterial count with the parity, indicating an impairment of the udder epithelial tissue, as well as a greater susceptibility to bacterial infections in older goats. The proximity of the udder to the floor was negatively affected by the SCC threshold, and needs to be taken into consideration for the udder health of dairy goats.

**Abstract:**

The effects of parity and somatic cell count in milk (SCC) threshold on the udder morphology, milkability traits, and milk composition was evaluated in 41 Canarian goats in mid-lactation. The animals were divided according to parity (1st, 2nd, and 3rd), and a SCC threshold of 2000 × 10^3^ cells/mL in milk was set to evaluate the effect of this factor on the different measured parameters. Results showed that primiparous goats had the udder smaller and less distended than multiparous goats, but no differences were detected on milk flow parameters. Furthermore, SCC and total bacterial count (TBC) tended to be higher when the parity increased. On the other hand, goats with SCC ≤ 2000 × 10^3^ had higher cistern-floor distance (CF) and lower TBC values compared with those goats with a count above the predetermined threshold. The results suggest that a reduction in SCC can be achieved by a selection of udder morphological traits. Moreover, milk flow parameters do not seem to be a tool to determine the udder health status in Canarian goats, but long-term studies are needed to verify it.

## 1. Introduction

In dairy animals, milk production and milking kinetics can vary depending on extrinsic factors such as milking machine characteristics, milking routine, and environmental conditions [1,2], along with intrinsic factors such as breed, stage of lactation, and parity [3,4]. In the case of parity effect, several studies have reported that the mammary gland of primiparous animals has a lower secretory capacity and a lower milk storage capacity compared with multiparous animals [5,6], and may affect milking flow rate parameters and milking duration.

Moreover, milk yield and milking speed depend also on udder anatomy [7]. In the case of small ruminants, they have proportionally larger cisterns on a relative basis than dairy cows, which play an important role in the storage of milk and can greatly affect the removal of milk at the time of milking [8]. Furthermore, udder conformation of many goat and sheep breeds is characterized by teats positioned more horizontally [9,10], a circumstance that implies manual intervention by the milker for a complete milk removal and affecting the milk ejection curve [11].

On the other hand, somatic cell count (SCC) of milk is a representative of the udder health and is widely used for evaluating milk quality [12]. In European countries, a legal threshold of 400 × 10^3^ cells/mL is set for raw cow’s milk [13]; in the US, it is 750 × 10^3^ cells/mL for cows and sheep, and 1500 × 10^3^ cells/mL for goats [14]. However, the European Union and most other countries around the world have yet to regulate SCC limits in small ruminant’s milk for various reasons related to sanitary control and quality aspects [15].

Somatic cells are a natural component of milk, and they comprise epithelial cells and white blood cells. It is well referenced that intramammary bacterial infections are the predominant cause of SCC increase [16]. However, apocrine milk secretion in goats and sheep implies higher amounts of epithelium cells and their fragments, arising from epithelial desquamation and physiological regeneration of mammary alveoli [17]. Furthermore, Canarian goats have a high proportion of machine stripping milk (around 20%) due to their udder morphology [18]. This milk is obtained by lifting the udder at the intramammary groove, while applying gentle downward traction to the teat cups, and then by brief manual massage of both udder halves [19]. This intervention may cause a higher epithelial desquamation, and therefore an increase in SCC in healthy animals.

Finally, several authors have studied the relationship between SCC and milkability traits in cows [20], sheep [21], and goats [7]. Nevertheless, the relation of these factors with the udder characteristics and milk quality is controversial. In this way, this trial aimed to study the effects of parity and SCC threshold on udder morphology, milkability traits, and milk composition during the mid-lactation period in Canarian goats. Goat production is an important economic resource in the livestock sector of the Canary Islands, where most of produced milk is used for the cheese industry. Therefore, knowledge about milk quality and safety parameters for goat milk may help to establish standards in the EU, considering the situation of the different regions that comprise it.

## 2. Materials and Methods

Animal procedures were conducted in strict accordance with the current European Animal Welfare Legislation (ART13TFEU).

### 2.1. Animals and Management

The present study was performed in the experimental farm of the Canary Islands Institute for Agricultural Research (ICIA, Tenerife, Spain) on 41 Canarian dairy goats in mid-lactation (106 ± 11 DIM). The animals were allocated into three groups according to parity (n = 14 in 1st parity, n = 13 in 2nd parity, and n = 14 in 3rd parity). The ration was formulated to cover requirements for lactating goats using INRAtion 4.0 software [22], and it consisted of commercial concentrate, corn grain, lucerne pellets, which were provided twice daily, and alfalfa hay offered for ad libitum consumption. Clean water and vitamin-mineral blocks were freely available for all animals.

Goats were milked in a double 12-stall parallel milking parlour (Alfa Laval Iberia SA, Madrid, Spain) equipped with recording jars (4 L ± 5%) and a low-line milk pipeline. Milking was performed at a vacuum pressure of 42 kPa, a pulsation rate of 90 pulses/min, and a pulsation ratio of 60/40 [23]. The milking routine included machine milking and stripping milking, performed by the same operator to remove the remaining milk from the udder before cluster removal, and teat dipping in an iodine solution (P3-cide plus; Henkel Hygiene, Barcelona, Spain). All animals were observed daily by an animal technician in order to detect any signs of disease.

### 2.2. Data Collection

During a 12-week period, data from milk flow parameters were recorded every two weeks, using a LactoCorder^®^ device (WMB, AG, Balgach, Switzerland) in the long milk tube. The variables computed were: total milk yield (MGG, kg), duration of total milking (tMGG, min), maximal milk flow rate (HMG, kg/min), amount of milk within the first minute (1 MG, kg), average milk flow (AvMF, MGG/tMGG, kg/min), and time to reach 500 g (tS500, min).

Milk samples (50 mL) were analysed immediately after collection to determine milk composition, SCC, and total bacterial count (TBC). Fat, protein, lactose, and total solids (TS) percentages were determined using a MilkoScan Mars^®^ (Foss Iberia S.A, Barcelona, Spain), SCC using a DeLaval Cell Counter^®^ (DeLaval International AB, Tumba, Sweden), and TBC using a BactoScan™ (Foss Iberia S.A., Barcelona, Spain).

Udder morphology measurements of each goat were taken just before the first (week 1) and the last milking (week 12) of the experimental period, and were taken in the stalls of the milking parlour before the pre-milking routine. The variables measured were: udder length (UL, cm), udder circumference (UC, cm) (both measured with a flexible tape), teat length (TL, cm), cistern-floor distance (CF, cm), and teat- floor distance (TF, cm) (measured with a rigid tape), on both halves. Criteria and measurement method were based on [10].

### 2.3. Statistical Analysis

An SCC threshold of 2000 × 10^3^ cells/mL in milk was set to evaluate the effect of this factor on the different measured parameters. Thus, two groups were formed according to the means of SCC recorded during the experiment: goats with SCC ≤ 2000 × 10^3^ cells/mL (n = 22, of which 11 were primiparous and 11 were multiparous) and goats with SCC > 2000 × 10^3^ cells/mL (n = 19, of which 3 were primiparous and 16 were multiparous). This threshold was established in accordance with previous studies, where milk SCC from goats free of intramammary infections ranged between 270 and 2000 × 10^3^ [24], while milk SCC from infected goats ranged between 2000 and 4000 × 10^3^ [25].

All statistical estimations were determined using SPSS 15.0 software (SPSS Inc., Chicago, IL, USA). The normal distribution of all variables was assessed using the Shapiro–Wilk normality test. Data of SCC and TBC were calculated and expressed as a logarithmic scale (log10) to achieve normal distribution. Parity effect was evaluated using a multiple comparison test by the Tukey’s post hoc method, while SCC threshold effect was assessed using a *t*-test. Statistical differences were considered significant at *p* < 0.05. Data are presented as means ± standard deviations.

## 3. Results

### 3.1. Parity

The effects of parity on udder morphological measurements, milk flow parameters, and milk quality are shown in Table 1. Parity significantly affected all udder morphological measurements. Thus, primiparous goats exhibited lower values of UL, UC, and TL respect to multiparous goats (*p* < 0.05), indicating that their udders are smaller. Furthermore, primiparous animals had higher values for CF and TF than multiparous animals (*p* < 0.05), evidencing that udder suspension becomes weaker with increasing age of the animals. No statistical significances were detected on milk flow parameters, nor on fat, protein, and TS percentages in milk. Nevertheless, the milk lactose fraction was higher in primiparous goats compared with multiparous goats (*p* < 0.05). Finally, SCC and TBC values tended to be higher when the parity increased, thus goats in first parity had significantly lower SCC and TBC than goats in third parity.

### 3.2. SCC Threshold

The effects of SCC threshold on udder morphological measurements, milk flow parameters, and milk quality are shown in Table 2. No significant differences were found for UL, UC, and TF measurements due to the SCC threshold factor, but goats with a count of ≤2000 × 10^3^ cells/mL in milk had lower and higher values for TL and CF, respectively, than goats with a count of >2000 × 10^3^ cells/mL in milk. Moreover, milk flow parameters and milk composition were not affected by the SCC threshold factor (*p* ≥ 0.05). However, TBC value was significantly higher in goats with a count above of 2000 × 10^3^ cells/mL in milk compared with those goats with a count below the predetermined threshold.

## 4. Discussion

### 4.1. Parity

Several studies have shown that udder morphology traits are significantly influenced by parity in dairy animals, which is consistent with the present findings. Third-parity goats had greater UL and UC and lower CF and TF compared with first-parity goats, which reflects an enlargement of the mammary cistern with increasing age. It has been reported that multiparous animals have larger cisterns than primiparous animals (in cows [26], ewes [27], and goats [5]). In the first lactation, the mammary gland of dairy animals is still developing and it tends to undergo modifications achieving a more well-developed and balanced structure during the subsequent lactations [28,29].

It is well known that cistern size plays an important role in the milk yield of dairy animals [9], and multiparous animals have a higher cisternal capacity than primiparous animals [5]. However, no significant differences in milk production were found among first-, second- and third-parity goats in the present study. Ref. [30] did not find differences in milk yield between primiparous and multiparous goats, and inferred that Canarian primiparous goats may have an optimal intramammary compliance and cisternal cavities. In any case, the similar milk yields between parities may be due to genetic factors reflecting an acceptable milk production level for the first-parity animals in mid-lactation.

Although in the present study milkability traits were not significantly affected due to parity factor, it has been reported that number of parity influences most of milk flow parameters. In dairy cows, Refs. [31,32] found that primiparous animals had a lower HMG and tMGG compared with older animals. Ref. [33] suggested that the cistern of primiparous animals remains full throughput milking due to its relatively constrained capacity, so milk is continuously resupplied from the alveoli, which combined with a smaller teat than that of multiparous animals, results in a more uniform curve and lower maximum milk flow rate.

On the contrary, Refs. [11,34] reported that AvMF and HMG had a decreasing trend with parity number in dairy ewes. These authors explained that milkability worsens because of the progressive deterioration of the degree of udder suspension with the age of the ewes. In agreement with the present results, Refs. [35,36] showed no significant differences in AvMF in dairy goats. It should be noted that Canarian goats store approximately 80% of total milk in the cisternal compartment [18], which may have minimized the parity effect on milk flow parameters.

In dairy goats, it has been reported that the lactose content of milk decreased with parity [37,38], which is in accordance with the present results. Ref. [30] found that multiparous goats had lower concentrations of milk lactose than primiparous goats upon milk stasis, and hypothesized it may be due to lactose passing from milk into blood through impaired tight junction. Ref. [39] interpreted this decline in milk lactose level in dairy ewes as the result of changes in the endocrine ± metabolic status as the number of lactations advances.

The positive association between SCC and parity in goats has been described in several studies [24,40,41]. This was confirmed in this study with a significant increase from first parity to third parity. Ref. [42] explained that the history of exposure to udder pathogens may partly explain the parity effect in goats because older animals may have chronic changes in the udder epithelial tissue, leading to having higher SCC. Previously, Ref. [43] found no evidence of this effect in goats for halves infected by minor and major pathogens where alterations of SCC caused by bacterial infection masked the effect of parity.

### 4.2. SCC Threshold

The SCC threshold group had a significant effect on some udder morphological measurements such as TL and CF. In dairy goats, Ref. [44] found that the udder shape, symmetry, degree of suspension, and degree of separation parameters showed to be different depending on SCC. These authors revealed that udder depth, and therefore its proximity to the floor, was different between the groups with low SCC (<1300 × 10^3^ cells/mL in milk) and high SCC (>6000 × 10^3^ cells/mL in milk), meaning that more attached udders presented lower SCC. This agrees with the findings of Ref. [45], who found that somatic cell score was genetically correlated with udder floor position in Alpine and Saanen breeds, and with teat length, teat width, and teat form in the Saanen breed. Additionally, Ref. [46] concluded that dairy ewes with a more horizontal teat position and larger teats had higher SCC, since they are more prone to develop subclinical mastitis. Therefore, the present results suggest that a reduction in SCC can be achieved by a selection of udder morphological traits.

Moreover, milk flow parameters were not significantly affected by the SCC threshold factor. This is in agreement with previous findings in cows [47] and ewes [21], where different SCC levels were set for each species. However, it has been reported that the shape of the milk ejection curve in dairy cows can be related to SCC levels, so the milk flow curve could give some information about udder health [48]. Thus, Ref. [31] found that from low SCC to high SCC there was a significant decrease in milk yield, time of plateau phase, and time of decline phase, and an increase in the maximum flow rate. In dairy goats, several studies also found that high SCC negatively influenced milk production and suggested the need for SCC monitoring in goat farms [49,50]. In this connection, Ref. [51] reported the existence of a positive genetic correlation between milk flow rate and SCC in Alpine and Saanen goats, indicating that the selection of animals with high milk flow would lead to deterioration in the udder health status.

Milk composition was also not significantly affected by the SCC threshold factor. Likewise, Ref. [21] found no significant effect of three SCC levels on content of fat, lactose, and TS in milk of dairy ewes, and Ref. [52] highlighted that milk composition did not change when milk SCC varied from 214 × 10^3^ to 1450 × 10^3^ cells/mL in milk in Alpine goats without evidence of clinical mastitis. On the other hand, Ref. [53] reported that the fat and lactose contents in the sheep milk of the higher SCC group (>1000 × 10^3^ cells/mL in milk) were lower than that of the lower SCC group (<1000 × 10^3^ cells/mL in milk), while protein and casein contents were not affected by the SCC level. Furthermore, Ref. [49] showed that goats with a higher SCC registered lower fat content and higher protein content in milk. Ref. [17] was able to show the evidence for changes in milk quality with SCC > 600 × 10^3^ cells/mL, and recommended that it should be routinely screened by dairy manufacturers to assure the consumer of high end-product quality.

Finally, it has been confirmed the positive relationship between SCC and TBC in Canarian goats. Ref. [54] reviewed that SCC is an indicator of the goat udder health and was strongly associated with bacterial growth in milk samples. In this way, Ref. [38] indicated that the lowest total SCC was found in milk samples free from any pathogens or with low number of environmental bacteria. Consistent with the above mentioned, Ref. [55] showed that total bacterial count is significantly correlated with the number of SCC in bulk milk. Nevertheless, Ref. [56] reported that no udder pathogens were found in goats with a high SCC (>1500 × 10^3^ cells/mL in milk), and positive results for udder pathogenic bacteria were obtained in goats with low SCC (≈850 × 10^3^ cells/mL in milk). Ref. [38] also showed that the bacterial pathogens were present in about 20% of goat milk samples containing low SCC (<1000 × 10^3^ cells/mL in milk). In addition, Ref. [57] suggested that the SCC and especially TBC in raw goat milk are at relatively low levels when strict selection against mastitis, regular health checks, and strict adherence to milking hygiene are applied. Therefore, there is still controversy regarding whether the SCC is a good indicator of bacterial infection of the mammary gland in goats.

## 5. Conclusions

According to our findings, there was an enlargement of the mammary gland with increasing age of animals, evidencing that udder suspension becomes weaker. However, this fact was not reflected in an increase of the milk yield among first-, second-, and third-parity goats. Surprisingly, milk flow parameters were not affected between parities, suggesting that the greatest milk storage capacity in the cisternal compartments of Canarian goats may have a minimal effect on milk flow emission. Furthermore, the positive association between SCC and TBC with the parity is clear, indicating an impairment of the udder epithelial tissue as well as a greater susceptibility to bacterial infections in older goats. On the other hand, it is noteworthy that the proximity of the udder to the floor needs to be taken into consideration not only for the suitability of dairy goats for machine milking, but also for the udder health due to higher SCC and TBC values. Finally, it is not altogether certain that milk flow parameters may be used as a tool to determine the SCC threshold and help to determine the udder health status in Canarian goats. Nevertheless, long-term studies are needed to verify it.

## Figures and Tables

**Table 1:** Effect of parity on udder morphological measurements, milk flow parameters, and milk quality.

	Parity			*p* Value
	1	2	3	
Udder morphological measurements				
UL, cm	25.36 ± 3.65 ^a^	31.08 ± 2.62 ^b^	29.21 ± 4.39 ^b^	0.001
UC, cm	60.82 ± 5.64 ^a^	67.08 ± 5.99 ^b^	67.86 ± 6.53 ^b^	0.007
TL, cm	3.84 ± 0.66 ^a^	4.84 ± 0.78 ^b^	4.28 ± 0.87 ^ab^	0.008
CF, cm	19.92 ± 2.56 ^b^	17.66 ± 4.70 ^ab^	15.54 ± 4.26 ^a^	0.020
TF, cm	26.69 ± 2.95 ^b^	22.32 ± 5.04 ^a^	21.52 ± 4.14 ^a^	0.004
Milk flow parameters				
MGG, kg	2.47 ± 0.51	3.00 ± 0.65	2.75 ± 0.74	0.104
tMGG, min	3.02 ± 1.00	3.49 ± 0.97	3.69 ± 1.47	0.306
AvMF, kg/min	0.87 ± 0.24	0.91 ± 0.27	0.79 ± 0.17	0.376
HMG kg/min	1.11 ± 0.25	1.17 ± 0.35	1.06 ± 0.22	0.584
1 MG, kg	0.91 ± 0.22	0.98 ± 0.31	0.87 ± 0.23	0.541
tS500, min	0.12 ± 0.49	0.11 ± 0.49	0.12 ± 0.30	0.770
Milk quality				
Fat, %	4.67 ± 0.47	4.60 ± 0.48	5.12 ± 0.78	0.059
Protein, %	4.32 ± 0.32	4.20 ± 0.32	4.30 ± 0.25	0.534
Lactose, %	4.53 ± 0.19 ^b^	4.30 ± 0.18 ^a^	4.34 ± 0.22 ^a^	0.009
TS, %	14.16 ± 0.59	13.76 ± 0.56	14.37 ± 0.79	0.065
SCC, log_10_ cells/mL	5.96 ± 0.28 ^a^	6.15 ± 0.42 ^ab^	6.30 ± 0.34 ^b^	0.045
TBC, log_10_ CFU/mL	4.32 ± 0.30 ^a^	4.67 ± 0.45 ^ab^	4.79 ± 0.51 ^b^	0.019

UL, udder length; UC, udder circumference; TL, teat length; CF, cistern-floor distance; TF, teat-floor distance; MGG, milk yield; tMGG, duration of total milking; AvMF, average milk flow; HMG, maximal milk flow rate; 1 MG, amount of milk within the first minute; tS500, time to reach 500 g; TS, total solids; SCC, somatic cell count; TBC, total bacterial count. ^a,b^ Means with different superscripts within the same row are significantly different (*p* ˂ 0.05).

**Table 2:** Effect of SCC threshold on udder morphological measurements, milk flow parameters, and milk quality.

	SCC Threshold		*p* Value
	≤2000 × 10^3^ Cells/mL	>2000 × 10^3^ Cells/mL	
Udder morphological measurements			
UL, cm	28.14 ± 4.16	28.89 ± 4.53	0.580
UC, cm	65.34 ± 7.15	65.05 ± 6.41	0.893
TL, cm	3.97 ± 0.68	4.70 ± 0.90	0.005 **
CF, cm	19.04 ± 3.98	16.17 ± 4.11	0.029 **
TF, cm	24.62 ± 5.48	22.28 ± 3.08	0.108
Milk flow parameters			
MGG, kg	2.64 ± 0.70	2.85 ± 0.60	0.312
tMGG, min	3.11 ± 0.70	3.73 ± 1.52	0.092
AvMF, kg/min	0.87 ± 0.20	0.85 ± 0.26	0.777
HMG kg/min	1.11 ± 0.21	1.11 ± 0.34	0.996
1 MG, kg	0.91 ± 0.20	0.93 ± 0.30	0.784
tS500, min	0.12 ± 0.42	0.12 ± 0.43	0.880
Milk quality			
Fat, %	4.88 ± 0.60	4.71 ± 0.66	0.393
Protein, %	4.27 ± 0.28	4.28 ± 0.33	0.954
Lactose, %	4.45 ± 0.17	4.33 ± 0.26	0.085
TS, %	14.23 ± 0.70	13.95 ± 0.66	0.190
SCC, log_10_ cells/mL	5.91 ± 0.29	6.40 ± 0.26	0.001 ***
TBC, log_10_ CFU/mL	4.33 ± 0.21	4.89 ± 0.50	0.001 ***

UL, udder length; UC, udder circumference; TL, teat length; CF, cistern-floor distance; TF, teat-floor distance; MGG, milk yield; tMGG, duration of total milking; AvMF, average milk flow; HMG, maximal milk flow rate; 1 MG, amount of milk within the first minute; tS500, time to reach 500 g; TS, total solids; SCC, somatic cell count; TBC, total bacterial count. ** *p* ˂ 0.01 and *** *p* ˂ 0.001.

## Data Availability

Data are contained within the article.
